# Secondary prevention for hepatocellular carcinoma in patients with chronic hepatitis B: are all the nucleos(t)ide analogues the same?

**DOI:** 10.1007/s00535-020-01726-3

**Published:** 2020-09-24

**Authors:** Terry Cheuk-Fung Yip, Jimmy Che-To Lai, Grace Lai-Hung Wong

**Affiliations:** 1 Department of Medicine and Therapeutics, 9/F Prince of Wales Hospital, 30-32 NganShing Street, Shatin, Hong Kong SAR, China; 2 Medical Data Analytic Centre (MDAC), Hong Kong SAR, China; 3 Institute of Digestive Disease, Faculty of Medicine, The Chinese University of Hong Kong, Hong Kong SAR, China

**Keywords:** Cirrhosis, Entecavir, Tenofovir disoproxil fumarate, Tenofovir alafenamide

## Abstract

Reducing the incidence of hepatocellular carcinoma (HCC) in patients with chronic hepatitis B (CHB) is the key ultimate goal set in essentially all treatment guidelines. There has been solid evidence supporting the relationship between serum hepatitis B virus (HBV) DNA level and risk of HCC. Antiviral treatment with oral nucleos(t)ide analogues (NAs) leads to sustained viral suppression and hence is often adopted as the secondary prevention for HCC in CHB patients. The first-generation NA, lamivudine, reduced the risk of HCC at 3 years compared to placebo; yet, its high emergence of antiviral resistance has made it no longer recommended in the international guidelines. Recent heated debate is about the two current first-line NAs—entecavir and tenofovir disoproxil fumarate (TDF)—Are they just as good to reduce HCC risk in CHB patients? A handful of cohort studies show two different kinds of observations—TDF is better than entecavir in lowering HCC risk, or these two NAs have led to similarly low risk of HCC. Tenofovir alafenamide (TAF), a modified version of TDF higher rate of ALT normalization, would be another potent nucleotide analogue is the treatment of choice for secondary prevention for HCC.

## Introduction

Hepatocellular carcinoma (HCC) is a major global health problem because of its high incidence rate and unfavorable clinical course [1]. In 2018, it was the sixth commonest cancer worldwide, with incidence of more than 841,000 cases/year, and the fourth leading cause of cancer-related deaths, with an estimated 781,000 deaths/year [2]. While HCC has a diverse etiology, chronic hepatitis B virus (HBV) infection is the key determinants in the most high-risk HCC areas (China, Eastern Africa) [2]. Although the risk factors for HCC development are well-known and great advances have been made through HBV vaccinations as primary prevention for HCC, the overall incidence and mortality rates of HCC are still rising.

HCC prevention can be categorized into three levels—primary, secondary and tertiary [3]. Primary prevention of HBV-related HCC is now feasible through HBV vaccination since early 1980s. By the end of 2016, 186 countries had introduced the HBV vaccine into their national immunization schedules [4], with many countries achieving greater than 80% coverage for the full recommended dose. The vaccine has dramatically reduced the prevalence rates of chronic HBV infection and the incidence of HCC at younger ages in high-risk countries in East Asia, where universal vaccination was first introduced [5]. While such primary prevention for HCC has benefited children and young adults, patients who were born before the availability of HBV vaccination and hence chronically infected with HBV cannot benefit from such primary prevention and remain at risk of developing HCC [6].

Recent developments in the antiviral treatment of HBV suggest that a significant proportion of HCC cases could be avoided [7]. The main treatment goal of chronic HBV infection is to reduce the risk of progression to cirrhosis and liver‐related complications, including HCC [8–10]. The current international treatment guidelines for patients with chronic hepatitis B (CHB) recommend entecavir, tenofovir disoproxil fumarate (TDF) and tenofovir alafenamide (TAF) as the first-line nucleos(t)ide analogues (NA) treatment. They have high antiviral effects and a high genetic barrier to drug resistance [8, 10]. Long-term entecavir and TDF treatment results in reduced incidence rate of HCC [11, 12]. Yet HCC risk is significantly reduced but not eliminated even in patients who have achieved prolonged complete viral suppression, as genetic and/or epigenetic aberrations with malignant potentials may have already accumulated in the background liver along with long-standing HBV infection [13].

An ongoing hot discussion in the field is whether one NA is better than the other in reducing HCC risk [14]. The first large-scale cohort study that compared the effectiveness of entecavir and TDF on reducing HCC was a Korean nationwide cohort study by Choi et al*.* They showed that TDF treatment was associated with a lower risk of HCC than entecavir treatment, and concordant result was shown in a hospital cohort [15]. Since then, there has been many other studies showing variable conclusions, with some of those studies coming from the same country but different observations [16]. In this review article, we will discuss the different approach as secondary prevention of HCC by antiviral treatment, with special focus on the efficacy of HCC risk reduction with different antiviral treatments.

### Viral load and HCC risk

High serum HBV DNA level is a well-known risk factor for HCC development among untreated CHB patients. In the REVEAL-HBV study, investigators prospectively followed 3653 hepatitis B surface antigen (HBsAg) carriers aged between 30 and 65 years in Taiwan for an average of 11.4 years [17]. Compared with participants who had serum HBV DNA of < 60 IU/mL, there was an independent dose-dependent relationship between elevated serum HBV DNA levels of ≥ 2000 IU/mL and HCC development adjusted for hepatitis B e antigen (HBeAg) status, alanine aminotransferase (ALT) level and presence of cirrhosis; the adjusted hazard ratio (HR) ranged from 2.3 (95% confidence interval [CI] 1.1–4.9) in patients with serum HBV DNA of ≥ 2000 to < 20,000 IU/mL, to 6.1 (95% CI 2.9–12.7) in patients who had serum HBV DNA ≥ 200,000 IU/mL. Also, an elevated serum HBV DNA level is associated with an elevated risk of progression to cirrhosis in a dose-dependent manner, while cirrhosis itself is a strong risk factor for HCC [18]. The strong relationship between serum HBV DNA level and HCC development is also reflected by its consistent presence in different untreated derived HCC risk scoring systems including REACH-B score [19], CU-HCC score [20] and GAG-HCC score [21]. Notably, 85% of the patients in the REVEAL-HBV study had negative HBeAg; the impact of high serum HBV DNA is more prominent among HBeAg-negative patients in the study, with an adjusted hazard ratio of 10.6 (95% CI 4.9–22.8) in patients who had serum HBV DNA ≥ 200,000 IU/mL as compared to those who had HBV DNA < 60 IU/mL. In fact, HBeAg-positive CHB patients who have very high serum HBV DNA (usually above 10^7^ IU/mL) and normal ALT are generally not at high risk of HCC development [22]. These patients are generally considered to have HBeAg-positive chronic HBV infection or previously known as immune-tolerant phase of CHB [8, 10]. In contrast, HBeAg-positive patients who have advanced age and moderately high HBV DNA of ≥ 20,000 IU/mL are at elevated risk of HCC [23].

Serum HBV DNA level is less discriminative on HCC development after the initiation of antiviral therapy in CHB patients. This is reflected by the good performance of treated derived HCC risk scores in the absence of HBV DNA including the PAGE-B-related scores [24–26] and CAMD score [27, 28]. The current first-line NA treatment has modified the natural history of CHB by a potent effect on HBV DNA suppression and a reduced risk of disease progression in both non-cirrhotic and cirrhotic patients [11, 12, 29]. The majority of treated patients can achieve complete viral suppression, i.e.*,* undetectable serum HBV DNA, on treatment [30, 31]. This is associated with a reduced risk of HCC [6, 31]. In contrast, CHB patients with persistent low-level viremia on treatment are associated with a higher risk of fibrosis progression and HCC development [32, 33]. On the other hand, HBsAg seroclearance is associated with a further HCC risk reduction on top of patients who achieved complete viral suppression on treatment [31, 34]. However, unlike complete viral suppression, HBsAg seroclearance remains uncommon in patients receiving the current first-line NA treatment.

### Evolving HBV treatment guidelines

There are three key, most widely adopted international guidelines issued by the authoritative international liver societies—namely the American Association for the Study of Liver Diseases (AASLD), Asian the Pacific Association for the Study of the Liver (APASL) and the European Association for the Study of the Liver (EASL)—outlining the framework and algorithms in management of CHB patients. With evolving evidence over the past decade, the guidelines have changed accordingly, yet by large they share similar recommendations over the years.

Taking APASL guidelines as examples, HBeAg-positive patients with HBV DNA > 20,000 IU/mL and ALT ≥ 2 times upper limit of normal (ULN) or evidence of moderate necroinflammation or fibrosis, judged by histological activity score or fibrosis stage, should receive antiviral treatment. For those with HBeAg-negative CHB, the threshold of HBV DNA is set at a lower cutoff at 2000 IU/mL. Until the latest two versions of APASL guidelines published in year 2012 and 2016, the use of noninvasive test of fibrosis with transient elastography has been advocated [9, 35]. Liver stiffness measurement (LSM) > 8.0 kPa would represent significant fibrosis, while LSM > 11.0 kPa would indicate cirrhosis in patients with normal ALT level. Patients with cirrhosis and detectable serum HBV DNA are indicated for antiviral treatment as well [9, 35].

There have been two main classes of antiviral treatment: interferon alpha and NA. Interferon alpha in the form of pegylated interferon alpha (PegIFN alpha) provides both direct antiviral and immunomodulatory actions, and hence has an advantage of finite treatment duration with no risk of resistance [36]. The drawbacks of PegIFN alpha include its multiple side effects and the need of subcutaneous injection. Furthermore, its use is not recommended in patients with decompensated cirrhosis and severe exacerbation of CHB as it may precipitate hepatic decompensation resulting in fatal complications. On the contrary, oral NAs are generally safe despite the need for long-term treatment in far majority of the patients.

Six NAs classified into nucleoside analogues (lamivudine, telbivudine, entecavir) and nucleotide analogues (adefovir, tenofovir [TDF and TAF]) are approved for treatment of CHB. Over the past decade, all three international guidelines have recommended the use of entecavir and two formulations of tenofovir (TDF and TAF) as treatment of choice regardless of the severity of liver disease, given their high resistance barrier with predictable high long-term antiviral efficacy leading to undetectable HBV DNA, as well as favorable safety profile, in the majority of compliant patients. NAs with low resistance barrier, such as lamivudine, telbivudine and adefovir, are not preferred nowadays given the high emergence of antiviral resistance, leading to suboptimal virological response.

Both entecavir and TDF have been indiscriminately suggested by guidelines for HBV treatment, unless under special circumstances, such as prior history of resistance to lamivudine or other nucleoside analogues, or during pregnancy, that would favor TDF over entecavir given the possible cross-resistance profile and pregnancy category C regarding entecavir, respectively. Up till the recent few years with latest guidelines, TAF hits the headline due to its superior plasma stability than TDF leading to more effective active metabolite delivery to hepatocytes, allowing a lower dose to be used with similar antiviral activity, less systemic exposure and thus decreased renal and bone toxicity [10]. The EASL 2017 guideline suggested entecavir and TAF over TDF in patients aged > 60 years and with established or at risk of bone or renal disease [8]. TAF, however, has not yet been recommended in pregnancy due to the lack of human data [37]. Thus, in current practice, entecavir and tenofovir (TDF and TAF) remain the preferred mainstay of NA treatment in chronic HBV infection.

### HCC risk reduction by various NA

#### NA with low genetic barrier to resistance

The first important data to demonstrate the efficacy of NA treatment in HCC risk reduction were those from the Asian randomized controlled trial, which compared lamivudine vs. placebo in NA-naïve patients with cirrhosis or advanced fibrosis and active liver disease [38]. After an early trial termination (up to 5 years in the initial protocol) with a mean treatment duration of approximately 3 years, lamivudine reduced the HCC risk compared with placebo by 51% (HCC incidence 3.9% vs. 7.4%), offering a benefit of marginal statistical significance (hazard ratio 0.49; *p* = 0.047) [38]. This landmark study receives some criticism for such early termination, as the marginal benefit if HCC risk reduction would have been negated by the development of drug-resistant mutants if the drug had been continued [39].

Similar results were reported in other studies with older NAs with low genetic barrier to resistance. In a systematic review assessing mostly lamivudine (with some studies using adefovir dipivoxil or the combination of both NAs) vs. no treatment in NA-naïve CHB patients, HCC incidence rates over a follow-up of 4 years were reduced in treated patients (2.8%) compared with untreated patients (2.8% vs. 6.4%; *p* = 0.003) [40]. A more recent meta-analysis also reported HCC rates of 3.4% in lamivudine-treated vs. 9.6% in untreated CHB patients over a follow-up of 4 years [41]. Telbivudine is generally underrepresented in such long-term cohort studies as the proposed roadmap approach for telbivudine, i.e.*,* switching to an NA with high genetic barrier to resistance in case of suboptimal virological response [42], has limited the treatment duration of telbivudine in most studies [43].

Because of the high rates of drug-resistant mutations as well as common treatment emergent adverse events [44], these three NAs with low genetic barrier to resistance are no longer being recommended as the first-line antiviral treatment by the latest international treatment guidelines [8, 10].

#### NA with high genetic barrier to resistance (e.g., entecavir, TDF, TAF)

Entecavir, TDF and TAF are the three first-line NA treatments with high genetic barrier to resistance that are recommended by the international treatment guidelines [8, 10]. Entecavir is a nucleoside analogue, while TDF and TAF are both prodrug of tenofovir, which is a nucleotide analogue. Both entecavir and tenofovir (TDF and TAF) have minimal risk of drug resistance in NA-naïve patients; tenofovir also has a very low rate of drug resistance in NA-experienced patients [8, 10]. Compared to lamivudine, entecavir has a significantly better virological, biochemical and histological responses in patients without previous exposure to nucleoside analogues [45, 46]. TDF also shows a superior antiviral efficacy with a similar safety profile as compared to adefovir dipivoxil [47]. Under long-term entecavir or tenofovir therapy, patients continue to have histological improvement and regression of liver fibrosis and even cirrhosis [48, 49]. Long-term therapy also prevents disease progression and HCC development [29, 50–52]. In terms of safety profile, long-term use of entecavir is generally safe [53]. While long-term use of TDF has been associated with bone and renal toxicity in some patients, TAF was designed to have a greater plasma stability that allows a more efficient delivery of tenofovir to the liver cells. This also allows a lower orally administered dose of TAF than TDF and reduces the systemic exposure of tenofovir in the body. Thus, TAF preserves the antiviral efficacy of TDF with improved renal and bone safety [54]. Interestingly, under a comparable rate of virological response, TAF shows a higher proportion of ALT normalization than TDF [54]. The same phenomenon is also observed in patients switching from TDF to TAF [55]. The society is anticipating long-term follow-up data on TAF use to see whether this phenomenon has any clinical implications on histological response and reduction in liver-related outcomes including HCC.

Reducing the risk of progression to cirrhosis and liver‐related complications including HCC is the main goal in managing CHB patients. While the risk of HCC is significantly reduced under current NA treatment, the risk is, however, not eliminated. Currently, entecavir and TDF are equally recommended as the first-line treatment for treatment-naïve CHB patients. However, this recommendation has recently been challenged starting from the first study by Choi et al*.,* which showed a better HCC chemoprevention effect of TDF over entecavir in a Korean nationwide historical population cohort of 24,156 patients and a validation hospital cohort of 2701 patients [15]. Propensity score-matched analysis demonstrated that TDF was associated with a lower risk on HCC development than entecavir therapy with a HR of 0.68. Surprisingly, soon after this study was published, another multicenter study from South Korea was published with a different conclusion, although a significant overlap of their cohort with that of the nationwide cohort study by Choi et al*.* is expected [16]. Kim et al*.* reported their results of a cohort of 2897 CHB patients from four academic, tertiary hospitals. They showed by propensity score-matched analysis that there was no statistically significant difference between TDF and entecavir treatment on HCC risk, with an HR of 1.02. Thereafter, multiple observational studies and meta-analyses concerning the same clinical question on TDF vs. entecavir on HCC development in CHB patients have been published and continue to show that either TDF was associated with a lower risk of HCC than entecavir treatment, or TDF and entecavir treatment did not have a significant difference on the risk of HCC development (Table 1) [56, 57]. One of the largest studies was conducted by the authors and colleagues based on a territory-wide retrospective cohort of 1309 and 28,041 patients treated by TDF and entecavir monotherapy in Hong Kong, respectively [58]. We showed that TDF was associated with a lower risk of HCC than entecavir treatment, over a median follow-up time of 3.6 years, based on propensity score weighting and matching analyses. Negative control outcomes were also applied to assess the issues of unmeasured confounding, which did not find any significant residual bias.

**Table 1:** Summary of baseline characteristics and outcomes of studies comparing entecavir (ETV) and tenofovir disoproxil fumarate (TDF) treatment on risk of HCC development (Modified from Choi et al. [57] and Dave et al. [56]

Study (Year)	Data source, country (study period)	Study type	No. of patients (% Male)	Age, years^a^	No. (%) of HBeAg positivity	No. (%) of NA treatment-naïve (%)	No. (%) of liver cirrhosis	Follow-up time, months	No. (%) of HCC and HR of TDF vs. ETV on HCC with 95% CI	PSM variables^d^
Liaw YF et al. (2011) [71]	39 sites in Europe (17 sites), Canada (4), Singapore (4), Taiwan (5) and the USA (9) (Apr 2006–Dec 2008)	Phase 2, double‐blind, RCT	TDF: 45 (82.2)	52 (48–57)	14 (31.1)	17 (37.8)	45 (100)^b^	48 weeks	TDF = 3 (6.7%) vs ETV = 1 (4.5%); HR, N.A	N.A
			ETV: 22 (77.3)	54 (47–58)	7 (31.8)	9 (40.9)	22 (100)^b^	48 weeks		
Koklu S et al. (2013) [72]	18 centers, Turkey	Observational	TDF: 72 (75.0)	54.2 ± 10.5	9 (12.5)	N.A	72 (100)	21.4 ± 9.7	TDF = 2 (2.8%) vs ETV = 4 (5.2%); HR, 0.60; 95% CI, 0.11–3.28	N.A
			ETV: 77 (77.9)	52.4 ± 11.2	17 (22.1)	N.A	77 (100)	24.0 ± 13.3		
Batirel et al (2014) [73]	4 centers (two universities, one tertiary education and research center, and one state hospital), Turkey (January 2008–October 2013)	Observational	TDF: 90 (65.6)	43.3 ± 12.9	29 (32.2)	90 (100)	0 (0)	27.2 ± 15.4	TDF = 0 vs. ETV = 0	N.A
			ETV: 105 (78.1)	42.0 ± 11.2	36 (34.3)	105 (100)	0 (0)	33.0 ± 15.4	HR, N.A	
Goyal SK et al (2015) [74]	Banaras Hindu University Hospital, India (January 2007–January 2014)	Observational	TDF: 220	47.3 (24–65)	85 (38.6)	173 (78.6)	220 (100)	45 (12–68)	TDF = 6 (2.7%) vs ETV = 4 (2.2%); HR, 0.49; 95% CI, 0.14–1.72	N.A
			ETV: 180	48.1 (26–65)	70 (38.8)	137 (76.1)	180 (100)	36 (11–60)		
Wu et al. (2007) [75]	Chang Gung Memorial Hospital, Taiwan (TDF: October 2011 to January 2014) (ETV: January 2007 to January 2012)	Observational	(Entire) TDF: 106 (69.8)	(Entire) 47.1 ± 12.1	(Entire) 50 (47.2)	(Entire) 106 (100)	(Entire) 29 (27.4)	(Entire) 37.9 ± 7.2	(Entire) TDF = 7.7% at 48 months vs ETV = 6.7% at 48 months HR, 0.73; 95% CI, 0.26–2.05 Cirrhosis subgroup, TDF = 17.1% at 48 months vs ETV = 16.2% at 48 months HR, N.A	1, 3–5
			ETV: 313(73.5)	47.0 ± 12.3	172 (55.0)	313 (100)	94 (30.0)	49 ± 19.1		
			(PSM) TDF: 106 (69.8)	(PSM) 47.1 ± 12.1	(PSM) 50 (47.1)	(PSM) 106 (100)	(PSM) 29 (27.4)	(PSM) 37.9 ± 7.2	(PSM) TDF = 7.7% at 48 months vs ETV = 5.1% at 48 months HR, N.A	
			ETV: 212 (76.4)	46.3 ± 13.2	100 (47.2)	212 (100)	57 (26.9)	47.8 ± 19.1		
Kayaaslan et al. (2018) [76]	6 Different centers (3 university hospitals and 3 education and research hospitals), Turkey (June 2008–June 2014)	Observational	TDF: 86 (55.8)	42 (range 18–71)	41 (47.7)	86 (100)	0 (0)	18 (range 12–72)	TDF = 0 vs. ETV = 0	N.A
			ETV: 166 (71.0)	43 (range 18–81)	56 (33.7)	166 (100)	0 (0)	48 (range 12–72)	HR, N.A	
Kim YM et al. (2018) [77]	Kyung Hee University Hospital, Korea (July 2007–January 2017)	Observational	TDF: 112 (62.5)	49.3 ± 10.9	62 (55.4)	70 (62.5)	30 (26.8)	38.5 ± 9.2	TDF = 3 (2.7%) vs ETV = 13 (6.8%); HR, 0.67; 95% CI, 0.19–2.35	N.A
			ETV: 191 (60.7)	47.7 ± 12.3	116 (60.7)	165 (86.4)	53 (27.8)	66.6 ± 26.8		
Yu JH et al (2018) [78]	Inha University Hospital, Korea (January 2007–December 2015)	Observational	TDF: 176 (59.1)	49 (range 20–84)	104 (59.1)	176 (100.0)	77 (43.8)	33.6 (range 6.3–60.5)	TDF = 7 (4.0%) vs ETV = 31 (7.6%); HR, 1.39; 95% CI, 0.56–3.45	N.A
			ETV: 406 (67.0)	53 (range 18–84)	212 (52.2)	406 (100.0)	148 (36.5)	69.9 (range 6–119.4)		
Kim BG et al (2018) [79]	Ulsan University Hospital, Korea (January 2007–April 2017)	Observational	(Entire) TDF: 604 (60.1)	(Entire) 50 ± 11	(Entire) 376 (62.3)	(Entire) 604 (100.0)	(Entire) 267 (44.2)	(Entire) 33 (21–46)	(Entire) TDF = 14 (2.3%) vs. ETV = 40 (5.5%); HR, 0.74; 95% CI, 0.39–1.39 aHR, 0.60; 95% CI, 0.28–1.30 Cirrhosis subgroup, TDF = 14 (5.2%) vs. ETV = 36 (10.4%); aHR, 0.67; 95% CI, 0.30–1.49 (PSM cohort) TDF = 7 (2.0%) vs. ETV = 24 (6.8%); HR, 0.53; 95% CI, 0.22–1.25	1–15
			ETV: 721 (65.3)	52 ± 11	430 (59.7)	721 (100.0)	346 (48.0)	66 (36–88)		
			(PSM) TDF: 354 (62.7)	(PSM) 51 ± 11	(PSM) 223 (63.0)	(PSM) 354 (100)	(PSM) 156 (44.1)	(PSM) N.A		
			ETV: 354 (62.1)	51 ± 11	232 (65.5)	354 (100)	169 (47.7)	N.A		
Choi J et al (2019) [15]	Asan Medical Center, Korea (January 2010–December 2016) National registry of patients with CHB (NHIS), Korea (January 2010–December 2016)	Observational	(Entire) TDF: 1141(60.6)	(Entire) 48.1 ± 10.5	(Entire) 641 (56.2)	(Entire) 1141 (100)	(Entire) 653 (57.2)	(Entire) 32.0 (23–40)	(Entire cohort) TDF = 39 (3.4%) vs. ETV = 115 (7.4%); HR, 0.64; 95% CI, 0.45–0.93; aHR, 0.66; 95% CI, 0.46–0.96 Cirrhosis subgroup TDF = 35 (5.4%) vs. ETV = 107(11.4%); aHR, 0.64; 95% CI, 0.43–0.95 (PSM cohort) TDF = 31 (3.6%) vs. ETV = 61 (7.0%); HR, 0.68; 95% CI, 0.46–0.99 (Entire cohort) TDF = 394 (3.1%) vs. ETV = 590 (5.1%); aHR, 0.68; 95% CI, 0.59–0.77 (PSM cohort) TDF = 350 (3.2%) vs. ETV = 567 (5.2%); HR, 0.68; 95% CI, 0.60–0.78 Cirrhosis subgroup TDF = 206 (7.1%) vs. ETV = 338 (11.6%); HR, 0.67; 95% CI, 0.56–0.80	1–6, 8–10, 12–21 1–2, 5, 14–15, 22–24
			ETV: 1560(61.9)	49.2 ± 10.5	853 (54.7)	1560 (100)	935 (59.9)	48.0 (36–48)		
			(PSM) TDF: 869 (62.1)	(PSM) 48.8 ± 10.4	(PSM) 481 (55.4)	(PSM) 869 (100)	(PSM) 505 (58.1)	(PSM) 32.0 (22–40)		
			ETV: 869 (59.7)	48.8 ± 10.4	479 (55.1)	869 (100)	511 (58.8)	48.0 (35–48)		
			(Entire) TDF: 12,692(62.6)	(Entire) 48.6 ± 9.8	(Entire) N.A	(Entire) 12,692 (100)	(Entire) 3488 (27.5)	(Entire) 37 (30.1–43.5)		
			ETV: 11,464(62.6)	49.3 ± 9.8	N.A	11,464 (100)	2991 (26.1)	51 (37.3–57.0)		
			(PSM) TDF: 10,923 (62.6)	(PSM) 49.0 ± 9.8	(PSM) N.A	(PSM) 10,923 (100)	(PSM) 2919 (26.7)	(PSM) 37 (30.0–43.4)		
			ETV: 10,923 (62.3)	49.1 ± 9.8	N.A	10,923 (100)	2891 (26.5)	51 (37.7–57.3)		
Cai et al. (2019) [80]	14 Tertiary hospitals or university hospitals, China (January 2012–December 2015)	Double‐blind for 48 weeks, then an open trial for 96 weeks, RCT	TDF: 157 (75.8)	30.8 ± 8.8	157 (100)	157 (100)	0 (0)	36	TDF = 0 vs. ETV = 0	
			ETV: 158 (76.6)	31.0 ± 8.4	158 (100)	158 (100)	0 (0)	36	HR, N.A	
Kim SU et al (2019) [16]	Yonsei University Severance Hospital, Kyungpook National University Hospital, Korea University Anam Hospital, and CHABundang Medical Center, Korea (January 2012–December 2014)	Observational	(Entire) TDF: 1413(64.6)	(Entire) 48.8 ± 12.0	(Entire) 694 (49.1)	(Entire) 1413 (100)	(Entire) 411 (29.1)	(Entire) N.A	(Entire cohort) TDF = 102 (7.2%) vs. ETV = 138(9.3%); aHR, 0.98; 95% CI, 0.75–1.27 Cirrhosis subgroup TDF = 66 (16.1%) vs. ETV = 108(21.6%); aHR, 0.83; 95% CI, 0.61–1.14 (PSM cohort) HR, 1.02; 95% CI, 0.77–1.35	1–3, 5, 8–9, 13–15
			ETV: 1484(59.9)	48.2 ± 11.5	758 (51.1)	1484 (100)	499 (33.6)	N.A		
			(PSM) TDF: 1278(62.1)	(PSM) 48.2 ± 12.0	(PSM) 640 (50.1)	(PSM) 1278 (100)	(PSM) 400 (31.3)	(PSM) N.A		
			ETV: 1278(62.1)	48.6 ± 114	640 (50.1)	1278 (100)	394 (30.8)	N.A		
Gordon et al. 2019 [81]^e^	Longitudinal Chronic Hepatitis Cohort Study (CHeCS), USA	Observational	TDF: 407	48	N.A	164 (20.0) of all 822 patients	151 (18.4) of all 822 patients	48	TDF = 13 (3.2%) vs. ETV = 18 (4.3%); aHR for Asian, 0.70; 95% CI, 0.29–1.68 aHR for non-Asian, 1.87; 95% CI, 0.60–5.87	N.A
			ETV: 415	51	N.A			66		
Yip TC et al (2019) [58]	Territory-wide registry of patients with CHB (CDARS), Hong Kong, (January 2008–June 2018)	Observational	(Entire) TDF: 1309 (45.1)	(Entire) 43.2 ± 13.1	(Entire) 721 (55.1)	(Entire) 1309 (100)	(Entire) 38 (2.9)	(Entire) 33.6 (16.8–54)	(Entire cohort) TDF = 13 (1.9%) vs. ETV = 285(5.9%); aHR, 0.36; 95% CI, 0.16–0.80 (PSM cohort) aHR, 0.39; 95% CI, 0.18–0.84	1–6, 8–10, 12–16, 25–27
			ETV: 28,041(64.5)	53.4 ± 13.0	8317 (29.7)	28,041(100)	3822 (13.6)	44.4 (20.4–60)		
			(PSM) TDF: 1200(48.9)	(PSM) 44.4 ± 13.1	(PSM) 625 (52.1)	(PSM) 1200 (100)	(PSM) 37 (3.1)	(PSM) 33.6 (18–54)		
			ETV: 4636(48.9)	42.9 ± 12.7	(53.5)	4636 (100)	(3.6)	34.8 (18–55.2)		
Hsu YC et al (2019) [66]	19 Centers from 6 countries or Regions (USA, China, HK, Japan, Korea, and Taiwan) based on the REAL-B consortium database	Observational	(Entire) TDF: 700(65.1)	(Entire)^c^ 45.7 ± 0.5	(Entire) 208 (33.7)	(Entire) 700 (100)	(Entire) 131 (18.7)	(Entire) 38.7 (23.8–56.2)	(Entire cohort) TDF = 13 (1.9%) vs. ETV = 285(5.9%); aHR, 0.81; 95% CI, 0.42–1.56 Cirrhosis subgroup aHR, 0.68; 95% CI, 0.27–1.68 PSM cohort) TDF = 11 (2.1%) vs. ETV = 19(3.7%); HR, 0.77; 95% CI, 0.37–1.60; aHR, 0.89; 95% CI, 0.41–1.92	1–6, 13–14, 28–29
			ETV: 4837(68.8)	50.2 ± 0.2	1537 (33.0)	4837 (100)	1344 (27.8)	60 (39.6–60)		
			(PSM) TDF: 520(65.0)	(PSM)^c^ 44.9 ± 0.6	(PSM) 338 (65.0)	(PSM) 520 (100)	(PSM) 105 (20.2)	(PSM) 38.9 (23.9–57.7)		
			ETV: 520(68.1)	44.1 ± 0.5	354 (68.1)	520 (100)	107 (20.6)	60 (36.5–60)		
Lee SW et al (2019) [62]	Catholic University, Korea (February 2007–January 2018)	Observational	(Entire) TDF: 1439(58.4)	(Entire) 47.3 ± 11.2	(Entire) 823 (57.2)	(Entire) 1439 (100)	(Entire) 483 (33.6)	(Entire) 36.4 (N.A.-N.A.)	(Entire cohort) TDF = 50 (3.5%) vs. ETV = 84(5.3%); aHR, 0.97; 95% CI, 0.68–1.40 Cirrhosis subgroup aHR, 0.99; 95% CI, 0.66–1.48 (PSM cohort) TDF = 47 (3.4%) vs. ETV = 64(4.7%); HR, 1.03; 95% CI, 0.70–1.51; aHR, 1.08; 95% CI, 0.52–2.24	1–15, 17, 30–35
			ETV: 1583(58.5)	46.7 ± 11.8	974 (61.5)	1583 (100)	567 (35.8)	60 (N.A.-N.A.)		
			(PSM) TDF: 1370(58.2)	(PSM) 46.9 ± 11.1	(PSM) 807 (58.9)	(PSM) 1370 (100)	(PSM) 464 (33.9)	(PSM) N.A		
			ETV: 1370(58.8)	47.0 ± 11.8	814 (59.4)	1370 (100)	465 (33.9)	N.A		
Kim WR et al (2019) [65]^e^	IQVIA Pharmetrics PlusTM Claims dataset, USA (January 2006–September 2018)	Observational	TDF: 5903(56.0)	N.A	N.A	5903 (100)	463 (7.8)	17.9 (7.9–34.7)	TDF = 39 (0.7%) vs. ETV = 46(1.2%); aHR, 0.61; 95% CI, 0.39–0.94	N.A
			ETV: 3819(63.1)	N.A	N.A	3819 (100)	370 (9.7)	17.0 (8.0–32.2)		
Lee CJ et al (2019) [61]^e^	Taipei Veterans General Hospital, Taiwan (March 2007–April 2018)	Observational	TDF: 288(61.8)	54.1 (24.0–94.1)	75 (33.5)	N.A	39 (13.5)	33.6 (8.4–124.8)	TDF = 8 (2.8%) vs. ETV = 31(6.9%); aHR, 0.86; 95% CI, 0.39–1.91 Cirrhosis subgroup TDF = 2 (5.1%) vs. ETV = 28(19.7%); aHR, 0.29; 95% CI, 0.07–1.24	N.A
			ETV: 452(65.7)	53.0 (23.4–89.7)	122 (33.7)	N.A	142 (31.4)	37.2 (6–145.2)		
Chang KC et al (2019) [82]^e^	Kaohsiung Chang Gung Memorial Hospital & Linko Chang Gung Memorial Hospital, Taiwan (January 2008—March 2018)	Observational	(Entire) TDF: 216(75.0)	(Entire) 56.1 ± 11.6	(Entire) 41 (19.0)	(Entire) 216 (100)	(Entire) 216 (100)	(Entire) N.A	(Entire cohort) TDF = 19 (8.8%) vs. ETV = 138(20.4%); aHR, 0.59; 95% CI, 0.36–0.95 (PSM cohort) aHR, 0.56; 95% CI, 0.31–0.98	1,6,8,11
			ETV: 678(72.4)	59.4 ± 11.1	125 (18.4)	678 (100)	678 (100)	N.A		
			(PSM) TDF: 159(74.2)	(PSM) 58.6 ± 11.2	(PSM) 28 (17.6)	(PSM) 159 (100)	(PSM) 159 (100)	(PSM) N.A		
			ETV: 610(73.6)	58.7 ± 10.6	114 (18.7)	610 (100)	610 (100)	N.A		
Papatheodoridis GV et al. (2020) [63]	10 Centers from 6 countries (Greece, Germany, Italy, Turkey, Spain, and the Netherlands)	Observational	TDF: 1163(71.1)	53 ± 13	233 (20.0)	521 (44.8)	(31.6)	90 (N.A.-N.A.)	TDF = 93 (8.0%) vs. ETV = 51(6.6%); aHR, 1.00; 95% CI, 0.70–1.42	N.A
			ETV: 772(69.7)	52 ± 14	110 (14.2)	607 (78.6)	(22.0)	91.2 (N.A.-N.A.)		
Pol S et al. (2019) [64]^e^	ANRS CO22 HEPATHER cohort, France	Observational	TDF: 1075(68.5)	46.7 ± 14.4	N.A	520 (48.4)	N.A	N.A	aHR, 1.07; 95% CI, 0.45–2.54	N.A
			ETV: 885(73.2)	50.0 ± 13.7	N.A	567 (64.1)	N.A	N.A		

*aHR* adjusted hazard ratio, *CI* confidence interval, *ETV* entecavir, *HCC* hepatocellular carcinoma, *NA* nucleos(t)ide analogues, *N.A.* not applicable, *No.* numbers, *PSM* propensity score matching, *RCT* randomized controlled trial, *SEM* standard error or the mean, *TDF* tenofovir disoproxil fumarate

^a^Parentheses indicate interquartile ranges; otherwise, data are expressed as mean ± standard deviation

^b^All patients had decompensated cirrhosis

^c^Data are expressed as mean ± SEM

^d^Propensity score-matched variables; 1. age; 2. sex; 3. hepatitis B e-antigen; 4. hepatitis B virus (HBV) DNA; 5. cirrhosis; 6. alanine aminotransferase; 7. aspartate aminotransferase (AST); 8. albumin; 9. bilirubin; 10. creatinine; 11. Alpha fetoprotein; 12. international normalized ratio or prothrombin time; 13. platelet count; 14. diabetes mellitus; 15. hypertension; 16. ascites; 17. Child–Pugh score; 18. Chinese University HCC score; 19. guide with age, gender, HBV DNA, core promoter mutations and cirrhosis-HCC score; 20. platelet age gender B score; 21. risk estimation for HCC in chronic hepatitis B score; 22. socioeconomic status; 23. level of health care; 24. smoking; 25. renal replacement therapy; 26. hepatic encephalopathy; 27. calendar year of treatment initiation; 28. country of study centers; 29. hepatic decompensation; 30. AST-to-platelet ratio index; 31. fibrosis-4 index; 32. body mass index; 33. alcohol; 34. esophageal varix; 35. gamma glutamyl transferase

^e^Conference abstracts presented in the Liver Meeting 2019, American Association for the Study of Liver Diseases (AASLD), Boston, the USA

In view of the conflicting results, investigators tried to compare the two studies by Choi et al*.* and Kim et al.[15, 16]. One of the reasons that lead to the different conclusion can be the differences on patient selection and exclusion criteria [14]. For instance, patients with decompensated liver cirrhosis were not included in Kim’s study. This can lower the statistical power of the study as these group of patients have the highest risk of HCC development, while they can still benefit from entecavir and TDF treatment and may thus be included into the study [59]. On the other hand, investigators raised concern about the unexpected patterns of cumulative incidence curve in Choi’s study, which may reflect the difference in treatment duration and suggest some unmeasured differences between the two treatment groups that confounded the treatment effect as in other observational studies [14]. Possible unmeasured differences can be drug adherence, surveillance programs and unreported prior exposure to older NA. This is also partly reflected by the higher rate of changing therapy in patients treated with entecavir than TDF in Choi’s study [60]. In addition, it is also important to take into account the difference in the follow-up time in the two treatment groups. As entecavir was available to the market earlier than TDF, the length of follow-up of entecavir-treated patients was usually longer than that of TDF-treated patients in most of the previous observational studies (Table 1). This can impact the result in both ways as entecavir-treated patients would have longer time to develop HCC and get detected. In contrast, entecavir-treated patients might benefit from a longer treatment duration as their subsequent risk of HCC can decrease after several years of treatment [50]. Yet, this phenomenon only impacts studies with long enough follow-up.

There have been a handful of observational cohort studies comparing the efficacy of TDF versus entecavir in HCC prevention since the publications of the studies we just discussed. Two studies focused on Asian patients [61, 62] two large-scale studies involved European patients [63, 64]: one recruited American subjects [65] and one of the mixed ethnicities [66]. These studies concluded either both NAs are just as good, or TDF is better than entecavir in HCC prevention. Amidst all the controversies, two recent meta-analyses by Choi et al*.* and Dave et al*.* draw the similar conclusions that TDF may be associated with lower risk of HCC when compared to entecavir [56, 57].

A randomized controlled trial with long follow-up would be a better approach than observational studies or meta-analysis of observational studies to provide evidence, yet it is not likely to be conducted as a large sample size is expected to capture enough patients who develop HCC and provide adequate statistical power to test the hypothesis. The society is thus anticipating findings from high-quality observational cohorts with complete patient data on severity of fibrosis, virological and biochemical response, concomitant medical conditions, drug adherence and adherence to surveillance program. Based on the current evidence, it is still insufficient to consider any change to the current recommendation of TDF or entecavir monotherapy for treatment-naïve CHB patients. In the future, the focus on comparison on drug effectiveness and chemoprevention may also switch from TDF to TAF when more long-term data are available.

## Conclusion and future perspectives

Provided that the available studies, which are still evolving, are not concordant on this topic, the possible virological, biochemical and immunological plausibility of TDF is the choice of secondary prevention for HCC may need further elucidation. Virologically, TDF achieves more potential virological suppression [15, 58], and possibly better reduction in HBsAg levels, compared to entecavir. Biochemically, more TDF patients achieved ALT normalization, an established protective factor of HCC [67], at 1 year in the Korean nationwide cohort [15]. Immunologically, nucleotide analogues (including TDF and adefovir dipivoxil) induce higher serum interferon lambda-3 (IFN-λ3) levels than nucleoside analogues (including lamivudine and entecavir) [68]. IFN-λ has potent antitumor activity in murine models of hepatoma [69], which may explain partly the difference in the lower HCC risk in TDF-treated patients reported. All these biological plausibility should be further substantiated in other human cohorts before a widespread paradigm shift in selecting TDF over other NAs for treatment-naïve patients.

As the residual risk of HCC during long-term NA treatment remains the key complication affecting patient outcome, additional observational cohorts in homogenous patients with data on liver disease severity, virological response and adherence to surveillance would be pivotal to draw a definitive conclusion and impact on treatment guidelines [60]. Furthermore, long-term data of TAF, a modified version of TDF with better safety profile and higher rate of ALT normalization [55, 70], would further consolidate if this potent nucleotide analogue is the treatment of choice for secondary prevention for HCC.

## Abbreviations

ALT: Alanine aminotransferase
CHB: Chronic hepatitis B
CI: Confidence intervals
DM: Diabetes mellitus
HBeAg: Hepatitis B e antigen
HBsAg: Hepatitis B surface antigen
HBV: Hepatitis B virus
HCC: Hepatocellular carcinoma
INR: International normalized ratio
IQR: Interquartile range
SHR: Subdistribution hazard ratio
TDF: Tenofovir disoproxil fumarate
TAF: Tenofovir alafenamide
